# Control of Cation Permeation through the Nicotinic Receptor Channel

**DOI:** 10.1371/journal.pcbi.0040041

**Published:** 2008-02-15

**Authors:** Hai-Long Wang, Xiaolin Cheng, Palmer Taylor, J. Andrew McCammon, Steven M Sine

**Affiliations:** 1 Receptor Biology Laboratory, Departments of Physiology and Biomedical Engineering and Neurology, Mayo Clinic College of Medicine, Rochester, Minnesota, United States of America; 2 Howard Hughes Medical Institute, Department of Chemistry-Biochemistry and Pharmacology, University of California San Diego, La Jolla, California, United States of America; 3 Department of Pharmacology, Skaggs School of Pharmacy and Pharmaceutical Sciences, University of California San Diego, La Jolla, California, United States of America; Harvard University, United States of America

## Abstract

We used molecular dynamics (MD) simulations to explore the transport of single cations through the channel of the muscle nicotinic acetylcholine receptor (nAChR). Four MD simulations of 16 ns were performed at physiological and hyperpolarized membrane potentials, with and without restraints of the structure, but all without bound agonist. With the structure unrestrained and a potential of −100 mV, one cation traversed the channel during a transient period of channel hydration; at −200 mV, the channel was continuously hydrated and two cations traversed the channel. With the structure restrained, however, cations did not traverse the channel at either membrane potential, even though the channel was continuously hydrated. The overall results show that cation selective transport through the nAChR channel is governed by electrostatic interactions to achieve charge selectivity, but ion translocation relies on channel hydration, facilitated by a trans-membrane field, coupled with dynamic fluctuations of the channel structure.

## Author Summary

Communication between a cell and its environment relies on channel-forming proteins to provide a low energy pathway for ions to move in and out. Although channel-forming proteins are essential to all life forms, the atomic-scale mechanisms that enable ions to pass through the channel remain elusive due to the lack of experimental approaches to monitor the protein and ion in real time and at atomic resolution. A powerful alternative approach is molecular dynamics (MD) simulation based on the laws of physics applied to the increasing body of protein structures resolved at atomic resolution. Here we present all-atom MD simulations applied to the nicotinic acetylcholine receptor (nAChR) that initiates voluntary movement in skeletal muscle. By focusing on individual permeant cations, we find that selective cation translocation occurs in stages: cations are first selected through a series of oppositely charged residues within the protein vestibule leading to a narrow hydrophobic constriction, but then hydration of the narrow region and dynamic fluctuations of the protein enable the cation to pass through. The findings provide a general framework for understanding how ions are selected for transport based on charge, and how the dynamic interplay between water, the ion, and the channel protein enable rapid ion translocation through the broad class of channel-forming proteins with hydrophobic barriers.

## Introduction

Channel proteins circumvent the enormous energetic barrier to ion transport imposed by the cell membrane and are essential to all life forms. When a channel activates, permeant ions flow passively down their electrochemical gradients, changing the membrane potential and allowing communication between extra- and intra-cellular environments. The selectivity of ion transport depends on the type of channel, which can be non-selective, charge-selective or ion-selective, suggesting a diversity of mechanisms underlying transport. Present day atomic structures of ion channels allow unprecedented studies of ion transport using computational approaches. Here we use all-atom molecular dynamics (MD) simulations to study transport of single cations through the charge-selective channel of the nicotinic acetylcholine receptor (nAChR) from the motor endplate.

The nAChR is a hetero-pentamer of 250 kD, and contains an intrinsic channel pore that triggers flow of cations in response to nerve-released ACh. The channel selects for cations, mainly according to size, and is formed by α-helices from the second transmembrane domains (TMD2) of each of the five subunits. Selectivity for cations is achieved by anionic residues located on either side of the channel mouth [1] and in fenestrated structures in the cytoplasmic domain [2], which stabilize cations and concentrate them relative to bulk solution. Hydrophobic residues extending from TMD2 line the narrow region of the nAChR channel [3], suggesting that the hydration shell around the ion is maintained as the ion passes through. The availability of a 4 Å resolution structural model of the *Torpedo* nAChR [4] has brought the atomic-scale mechanism of ion transport under intensive investigation [5,6].

MD simulations of a simplified channel, composed of only TMD2 domains embedded in a bilayer-mimetic slab, revealed that although water filled the channel along its entire length, ions did not enter [7]. In simulations containing the entire pore domain in an explicit lipid bilayer, water and ions were excluded from the narrow region of the channel [8]. However, manually widening the pore radius by 1.5 Å allowed penetration of both water and ions, giving an ion transport rate approaching that expected from the single channel current amplitude. Although neither of these studies included the effect of membrane potential, they concluded that the cryo-electron microscopic structure of the *Torpedo* nAChR is in the non-conducting, inactive state.

Analogous MD simulations have been conducted on the mechano-sensitive channel of small conductance (MscS), which like the nAChR, also contains a hydrophobic pore. The initial simulations suggested that the x-ray structure of MscS was in the non-conducting state because water was excluded from the narrow hydrophobic region during the majority of the simulation [9]. However, application of a membrane potential promoted water entry, increased the channel radius, and produced ion transport at rates approaching those measured experimentally [10–12]. These later simulations suggest that membrane potential facilitates entry of water, promoting ion transport, and that the x-ray structure of MscS is closer to a conducting than a non-conducting state.

Here, we generate a homology model of the nAChR found at the human adult motor endplate, embed it in explicit lipids and solvate it with water and ions. We apply a trans-membrane electric field to mimic the cell membrane potential, and perform all-atom MD simulations with and without restraints of the protein structure. Our results reveal transport of single cations, and show that the transport depends on hydration of the channel, facilitated by a trans-membrane field, coupled with dynamic fluctuations of the channel structure.

## Results/Discussion

We first subjected the nAChR ensemble, including one copy of the receptor protein, membrane lipids, water, Na^+^ and Cl^−^ ions, to a 10 ns equilibration step (see Methods). Then we applied a voltage bias equivalent to a physiological trans-membrane potential of −100 mV, and generated a further MD simulation of 16 ns. Starting at 8 ns following application of the voltage bias, a series of snapshots reveal a single cation traversing the hydrophobic TMD2 region of the channel (Figure 1). The snapshots depict the cation and the water-filled volume within TMD2, and show temporal oscillations of the water volume that thins and widens at varying positions along the channel axis, suggesting cation transport is associated with water translocation.

**Figure 1:**
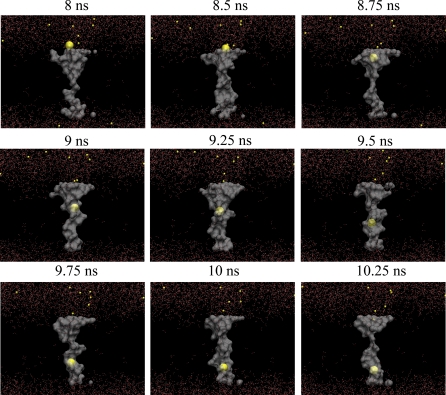
Successive Snapshots from the MD Simulation Show Translocation of a Single Cation through the nAChR Channel (Applied Trans-Membrane Potential, −100 mV) Water molecules inside the nAChR channel are shown in surface representation, with the permeating cation in VDW representation. Water molecules outside the channel are shown in line representation, and other cations are shown as small yellow spheres. The protein and lipids are omitted.

### Cation Trajectory through the Channel

With the simulation trajectory in hand, we tracked the position of the cation along an axis through the center of the channel and plotted position as a function of time (Figure 2). The cation exhibits step-wise position changes, with dwells at each position varying in duration. In the extracellular region bordering TMD2, dwells at each step are prolonged, whereas dwells within TMD2 are transient. The position of the prolonged dwell time immediately apical to TMD2 (upper gray arrow) corresponds to the “outer” ring of negatively charged residues previously shown to affect unitary conductance of the channel [1]. Further in the apical direction, two more positions of stability are detected (black arrows), corresponding to rings of polar or negatively charged residues within the lumen of the extracellular domain; the contributions of these more apical residues to unitary channel conductance have not been examined experimentally. Within TMD2, several positions are occupied transiently, corresponding to rings of hydrophobic side chains that line the channel and form the barrier to ion transport.

**Figure 2:**
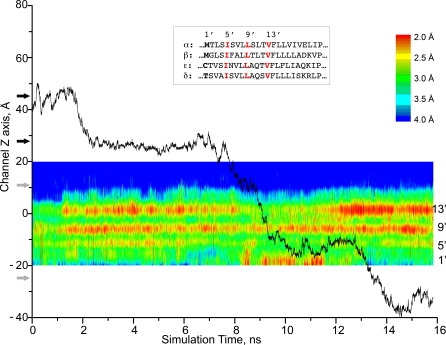
Position of the Cation Along the Channel Axis from the Simulation in Figure 1 Superimposed upon a Color-Encoded Plot of the Effective Pore Radius (See Calibration Scale), Measured Using the Program HOLE [30] The Z-axis position of the lipid membrane spans from −20 to 20 Å. Gray arrows indicate previously described extracellular and intracellular rings of charged residues [1], while the black arrows indicate two more rings of charge coinciding with prolonged dwell times of the cation. Residues of TMD2 that line the channel are indicated by consensus position numbers from intra-cellular to extra-cellular, 1′, 5′, 9′, and 13′.

Color-encoded changes in the effective pore radius along TMD2 are superimposed upon the time course of cation transport (Figure 2). Four horizontal stripes enriched in yellow and red colors indicate narrow regions corresponding to rings of hydrophobic side chains at the standard consensus positions 1′, 5′, 9′, and 13′ of TMD2 [13]. The pore radius corresponding to each of these rings oscillates during the simulation trajectory, but the radius at position 9′ shows much smaller oscillations. Until around 8 ns, the radius at the upper 13′ position is narrow, but it widens just before the cation approaches and it stays wide until the cation passes by. Concurrent with the widening at position 13′, the radius at position 1′ constricts even though the cation is far from this position. Next, the cation surpasses the central 9′ position with little change in the local pore radius, but it hops back and forth across position 5′ until the radius at position 1′ widens enough to allow complete translocation of the cation. Simultaneous with widening at position 1′, the more extracellular 13′ position constricts. Thus when the cation enters, the extracellular portion of TMD2 widens while the intracellular portion constricts, and when the cation exits the sequence of motions is reversed. The changes in channel radius reveal a back and forth tilting of TMD2 about an axis centered at the 9′ position that accounts for the peristaltic changes in water volume in Figure 1.

### Relationship between Channel Hydration and Cation Translocation

Next, we examined the cycles of emptying and filling of water within the channel by counting the number of water molecules in three contiguous zones, 6 Å thick, centered at positions 5′, 9′, and 13′ (Figure 3). At the beginning of the simulation, water occupancy is high throughout the channel, but 2 ns after application of the trans-membrane potential, water occupancy in the zones corresponding to positions 13′ and 9′ falls precipitously, creating a water-free volume known as a vacuum plug. However at 8 ns, just before the cation enters, water transiently refills these two zones for a 2 ns period that coincides with cation transport. Afterward, water diminishes in these two zones and no further cation transport occurs. Thus cation transport is associated with periods of channel hydration.

**Figure 3:**
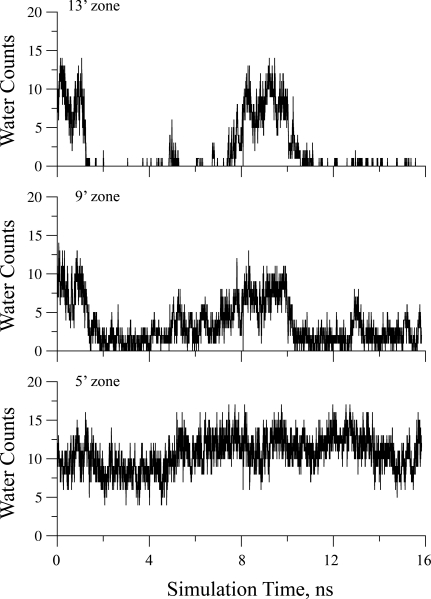
Number of Water Molecules in the Channel in Contiguous 6 Å Thick Zones Centered at TMD2 Positions 5′, 9′, and 13′ Plotted against Simulation Time (Trans-Membrane Potential, −100 mV)

### Contribution of Trans-Membrane Potential to Channel Hydration

To examine the contribution of the trans-membrane potential to cation transport, we started with the same equilibrated system and generated another 16 ns simulation with a hyperpolarized potential of −200 mV. The resulting trajectory shows that three cations achieve stable positions in the extracellular vestibule bordering TMD2, and that two of them traverse the channel (Figure 4). As observed at −100 mV, each cation moves in discrete steps, exhibiting both prolonged and transient occupancies in the course of transport. Four positions of prolonged occupancies are observed in the extracellular vestibule, three of which were seen in the simulation at −100 mV. The fourth stable position corresponds to yet another ring of negatively charged residues that lines the extracellular lumen in the vicinity of the ligand-binding domain (black arrow). A fifth stable position on the intracellular side of TMD2 is also evident in this trajectory (gray arrows), corresponding to the intracellular rings of charged residues previously shown to contribute to unitary channel conductance [1]. Although the hyperpolarized potential leads to an overall widening along the length of the channel (less yellow and red color in Figure 4), the radius again shows reciprocal changes at positions 1′ and 13′, consistent with tilting of the TMD2 helices about an intervening axis.

**Figure 4:**
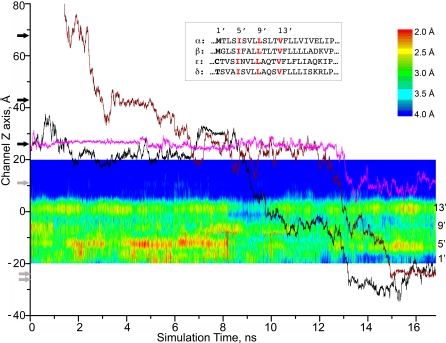
Positions of Three Cations along the Channel Axis from a Simulation with a Trans-Membrane Potential of −200 mV Superimposed upon a Color-Encoded Plot of the Effective Pore Radius, as in Figure 2 The Z-axis position of the lipid membrane spans from −20 to 20 Å. Gray arrows indicate previously described extracellular and intracellular rings of charged residues [1], while the black arrows indicate three additional rings of charge coinciding with prolonged dwell times of the cations.

The simulation at −200 mV also captures transport of two cations close together in time. While the first cation is traversing the channel, the second cation remains outside of TMD2, oscillating between two stable positions within the central vestibule of the ligand binding domain. Once the first cation exits the channel, the second cation enters. Meanwhile a third cation reaches the border of TMD2 only after the second cation enters the channel. Although the increase in transmembrane potential decreases the time between entry of successive cations, only a single cation at a time occupies the hydrophobic region between positions 1′ and 13′.

We noted an increase of 0.5 Å in the effective channel radius at −200 mV, corresponding to widening of the two zones centered at positions 13′ and 9′ (Figure 5). This relatively small change in radius is associated with substantial increases in water occupancy within these zones, and loss of the cycles of emptying and filling seen at −100 mV. In MD simulations of the MscS channel, similar increases in channel radius and water occupancy were observed when increasing transmembrane potentials were applied [10]. Our results for the nAChR and those for MscS suggest that water occupancy of the channel is the principal requirement for cation transport through a hydrophobic channel. However, the continuous hydration of the channel observed at −200 mV did not promote a commensurate increase in cation transport during the 16 ns simulation. Thus in addition to changes in channel radius and hydration, an additional kinetic limitation for cation translocation appears to be involved.

**Figure 5:**
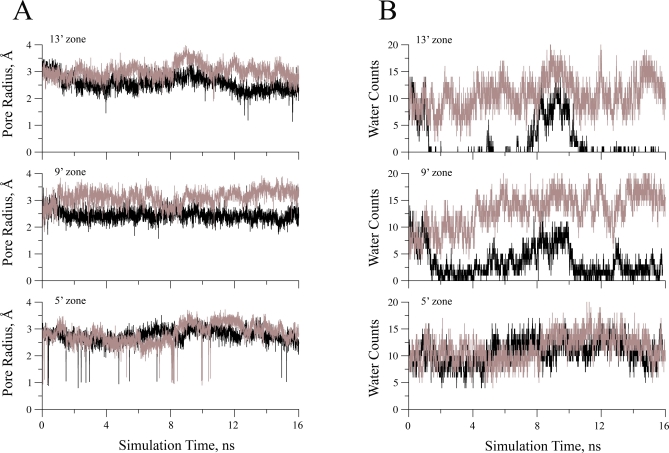
Pore Radius and Number of Water Molecules in Contiguous 6 Å Thick Zones Centered at TMD2 Positions 5′, 9′, and 13′ Plotted against Simulation Time Black traces represent the simulation at −100 mV, and the brown traces represent the simulation at −200 mV.

### Contributions of Dynamic Protein Fluctuations

The changes in water volume and channel radius shown in Figures 1 and 2 suggest that dynamics of the protein may be the additional kinetic limitation for cation transport. To examine the contribution of protein dynamics, we performed two additional MD simulations at each of the two trans-membrane potentials, but with restraints of the protein structure. No cation translocation was observed at either potential. At −100 mV, one cation entered the channel and approached the narrowest constriction, but it retraced its path by the end of the simulation (Figure 6). Parallel measurements of water occupancy reveal full hydration of the channel in all three hydrophobic zones, and show no differences in hydration between the two potentials (Figure 7). Thus, in addition to increases in channel radius and hydration, dynamic fluctuations of the protein structure are required for cation transport. Our overall simulations show that cation selectivity of the nAChR is governed by multiple electrostatic interactions between the cation and charged residues flanking the channel, but that subsequent translocation relies on channel hydration, facilitated by the trans-membrane potential, and dynamic fluctuations of the channel structure.

**Figure 6:**
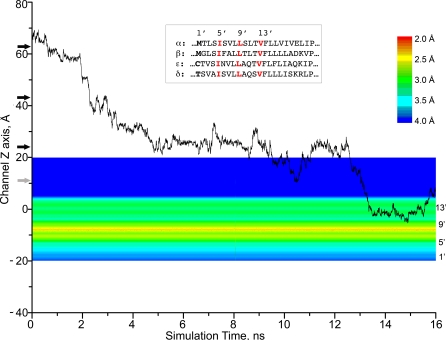
Position of a Cation along the Channel Axis from Simulation of the Restrained Structure at −100 mV Superimposed upon a Color-Encoded Plot of the Effective Pore Radius, as in Figures 2 and 4

**Figure 7:**
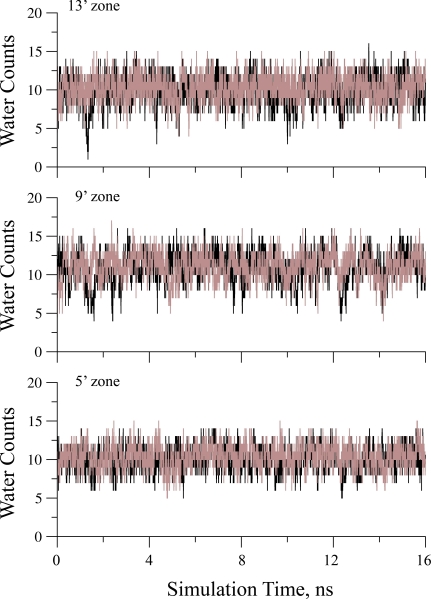
Number of Water Molecules in Three 6 Å-Layered Zones Centered at TMD2 Positions 5′, 9′, and 13′ Plotted against Simulation Time for the Restrained Structure Black traces represent the simulation at −100 mV, and the brown traces represent the simulation at −200 mV.

### Structural Bases of Cation Selectivity and Translocation

Our simulations reveal single cation translocation events through the channel of the human muscle nicotinic AChR. The findings shed new light on the nature of cation selectivity and on how single cations are transported one at a time across the central hydrophobic barrier. Four rings of polar or negatively charged residues line the central vestibule of the extracellular domain and likely contribute to cation selectivity. Although the narrowest hydrophobic pore is wide enough to accommodate a sodium ion with a single hydration shell, ion transport requires both hydration of the channel and dynamic motions of the protein. Continuous hydration of the channel is achieved by increasing the trans-membrane potential [14], which leads to widening of the channel and loss of the cycles of water emptying and filling of the narrow hydrophobic region. However, restraining the protein structure prevents ion transport even though the channel is continuously hydrated and the narrowest opening remains large enough for a sodium ion with its hydration shell. Dynamic motions during ion transport are associated with a back and forth tilting of TMD2 about the 9′ position and the peristaltic increases in the water-filled volume, some of which are associated with cation translocation across the central hydrophobic barrier. Thus ion transport through the nAChR requires a coordinated interplay between dynamic fluctuations of the protein, water and the permeating cation.

Our findings suggest that rings of negatively charged residues on both sides of TMD2 stabilize permeant cations. The functional significance of the rings immediately flanking TMD2, αGlu262, αGlu241 and αAsp 238, was originally uncovered by monitoring changes in unitary channel conductance following site directed mutations [1], and give confidence in the capability of our simulations to define structural counterparts of cation selectivity. Our findings suggest three more rings of functionally analogous polar or negatively charged residues within the central vestibule of the ligand binding domain, corresponding to αAsn 47, αGlu 83 and αAsp 97, together with residues at equivalent positions in the non-α-subunits (Figure 8). A series of rings along the external vestibule seems well suited to selecting cations for transport, as once a cation passes the first ring, it is further stabilized by additional rings within the lumen, thus increasing the cation concentration compared to bulk solution. This interpretation can be readily tested by mutagenesis coupled with electrophysiological measurements of unitary channel conductance and ion selectivity, and by x-ray crystallography of isolated ligand binding domains in the presence of suitable ions.

**Figure 8:**
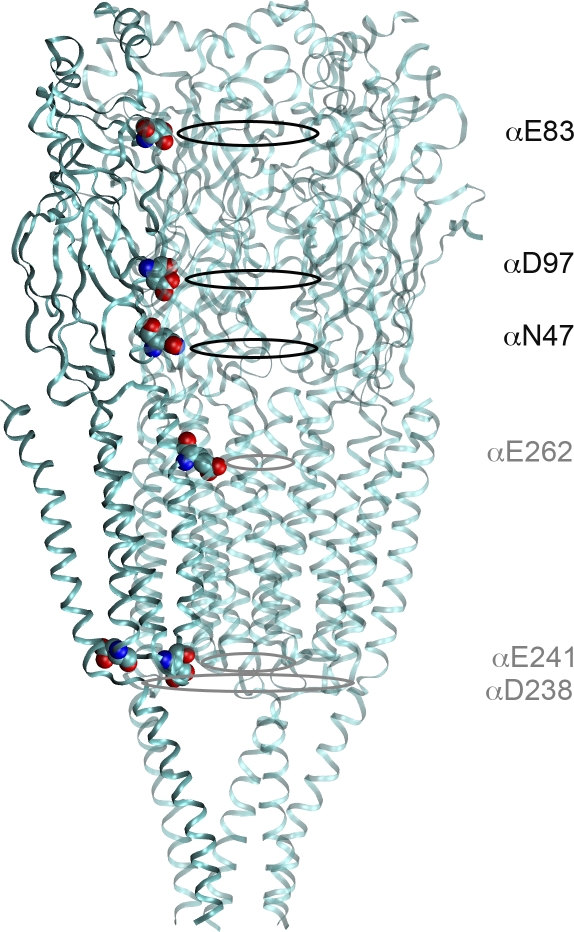
Homology Model of the Human Adult Muscle nAChR Used in the Simulations Rings of charged residues in the extracellular lumen flanking TMD2 are shown. Rings and labels in gray indicate previously described rings of charged residues that affect conductance and selectivity (1), whereas rings in black indicate newly identified residues associated with prolonged cation dwell times in Figures 2 and 4.

Once the cation reaches TMD2, hydrophobic interactions dominate the transport process. The rings of non-polar side chains lining TMD2 form a narrow constriction analogous to the hydrophobic channel of MscS [10–12] and hydrophobic nanotubes [15–18]. As we observe in the nAChR, these systems exhibit increases in hydration with only slight increases in channel radius, and the cycles of water filling and emptying diminish with increasing trans-membrane potential, likely through increasing the degree of order of the water molecules. The collective observations suggest that the switch from an inactive to an active channel could occur with increases in channel radius of one Å or less. The non-polar side chains along TMD2 provide little stabilization for a hydrated cation, minimally slowing transport, and suggest a steric interplay between the rings of non-polar side chains and the cation may limit transport in this region. The non-polar side chains also create an environment of low electric permittivity, extending the distance range of inter-cation Coulombic repulsion, perhaps explaining why only a single cation at a time occupied TMD2.

Our findings indicate that dynamic fluctuations of the narrow hydrophobic region of the nAChR channel are crucial for cation transport. The effects of dynamic fluctuations likely originate from multiple sources. First is the effect of dynamics on hydration of the channel. We find that although the restrained protein allows continuous hydration of the channel and maintains an opening wide enough for a hydrated sodium ion, transport is prevented. Similarly in model hydrophobic pores, rigid walls promote water filling and flexible ones favor water vapor states [17]. Second, computational studies of gramicidin suggest protein dynamics affects mobility of water and ions in a narrow pore where the magnitude of thermal fluctuations is significant relative to pore size [19]. Third, protein dynamics can affect the free energy barrier to ion translocation [31]. The potential of mean force (PMF) for a pore composed of rigid TMD2 helices from the *Torpedo* nAChR exhibited a peak for a sodium ion of 9 kcal/mol [7]. A much smaller peak of 3 kcal/mol was obtained in PMF calculations based on dynamically fluctuating TMDs from a homology model of the α7 nAChR [20]. Although the two reports employed different models of the nAChR channel and different methods for calculating the PMF, the overall findings suggest protein dynamics reduces the energetic barrier for ion translocation. The effect of protein dynamics on the PMF profile can be further addressed by calculations applied to the same nAChR channel under restrained and unrestrained conditions.

### Single Cation Translocation and Origin of Unitary Current

A natural question arises of why cation translocation is observed at all in our unrestrained simulations. Our nAChR structural model is expected to be in the inactive state because the structure of the modeling template, the *Torpedo* nAChR at a resolution of 4 Å, was obtained in the absence of agonist [4]. However, the inactive state may have a low but non-zero cation permeability. Single channel recording techniques register step changes in current between inactive and active states, but cannot resolve single ion translocation events due to limitations in bandwidth and background noise. Thus a low frequency of single ion translocation events may be inherent to channels with hydrophobic pores. Because a unitary current amplitude of 5 pico-amperes amounts to 4 or 5 cations transferred per 100 ns, simulations well beyond 16 ns are required to establish the functional state of the channel. Future MD simulations spanning much longer times are thus of high importance.

By focusing on the permeating cation, the present simulations show that selective ion transport arises from electrostatic interactions between the cation and multiple charged side chains in the protein, combined with dynamic interactions between the hydrophobic constriction, the cation and water. Although the functional state of the nAChR in our simulations is undetermined, the combination of dynamic fluctuations of the protein and cycles of water emptying and filling, facilitated by the transmembrane potential, likely govern cation permeation in both active and inactive receptor states.

## Methods

### Homology modeling.

We used the comparative protein structural modeling program, MODELLER [21], to generate a homology model of the adult human nAChR using the *Torpedo* structural model [4] as the template. The modeling procedures are described in previous papers [6,22,23]. The nAChR model was imbedded in a fully solvated lipid (POPC) bi-layer (120 Å × 120 Å) using the VMD *membrane* plug-in [24]. Lipids within 0.8 Å of the protein were removed. The total number of lipids was 298, with 141 on the extracellular side and 157 on the intracellular side. Next, the membrane-protein complex was solvated in TIP3P water using the VMD *solvate* plug-in. Ions were added to neutralize the net charge of the protein using the VMD *autoionize* plug-in, and amounted to 84 sodium and 26 chloride atoms, achieving a salt concentration of 100 mM. The resulting system comprises 247,568 atoms, which includes 1886 protein residues and 59,080 TIP3P water molecules.

### MD simulations.

We used the highly scalable molecular dynamics simulation program, NAMD [25], and the CHARMM27 force field [26]. Once the protein ensemble was built, the following four rounds of equilibrations were completed. (i) 2,000 steps of energy minimization for the non-backbone atoms. (ii) five cycles of a 500-step energy minimization with decreasing position restraints on the protein C^α^ atoms. (iii) a gradual increase in the temperature from 50 °K to 310 °K in 10,000 steps of constant volume (NVT ensemble ) simulation with restraints (with a force constant of 3 kcal · mol^−1^ · Å^−2^) applied to the protein C^α^ atoms. (iv) a 2 ns constant surface area ensemble MD equilibration with decreasing positional restraints on the C^α^ atoms. A short cutoff of 9 Å was used for non-bonded interactions, and long-range electrostatic interactions were treated using the Particle Mesh Ewald method [27]. Langevin dynamics and a Langevin piston algorithm were used to maintain the temperature at 310 °K and a pressure of 1 atm. The r-RESPA multiple time step method [28] was employed with a 2 fs time step for bonded atoms, a 2 fs step for short-range non-bonded atoms, and a 4 fs step for long-range electrostatic forces. The bonds between hydrogen and heavy atoms were constrained with the SHAKE algorithm. An external electric field was applied uniformly to all atoms of the system along the z-direction perpendicular to the membrane plane [29]. The voltage difference V across the simulated cell is determined by the product of the length of the simulated cell L_z_ and the uniform electrostatic field E_z_, both in the z-direction. All atoms of the nAChR protein were fixed during restrained simulations. All MD simulations were performed on a 64-processor Linux-cluster in the Receptor Biology Laboratory at Mayo Clinic.

## Abbreviations

MD: molecular dynamics
MscS: mechano-sensitive channel of small conductance
nAChR: nicotinic acetylcholine receptor
TMD2: second trans-membrane domain
